# The Role of Humoral Alloreactivity in Liver Transplantation: Lessons Learned and New Perspectives

**DOI:** 10.1155/2017/3234906

**Published:** 2017-01-09

**Authors:** Elaine Y. Cheng

**Affiliations:** ^1^Terasaki Foundation Laboratory, Los Angeles, CA, USA; ^2^Division of Liver and Pancreas Transplantation, Department of Surgery, University of California, Los Angeles, CA, USA

## Abstract

More than ten years after the initial description of the humoral theory of transplantation by Dr. Paul I. Terasaki, the significance of humoral alloimmunity in liver transplantation has yet to be clearly defined. The liver allograft has an inherent tolerogenic capacity which confers its resistance to cell-mediated as well as antibody-mediated rejection. Nevertheless, the protection against alloimmunity is not complete, and antibody-mediated tissue injury can occur in the liver graft under specific circumstances. In this article the evidence on the clinicopathologic effects of donor-specific alloantibodies in liver transplantation will be examined and interpreted in parallel with lessons learned from renal transplantation. The unique anatomic and immunologic features of the liver will be reviewed to gain new insights into the complex interactions between humoral immune system and the liver allograft.

## 1. Introduction

An increasing body of evidence has been published over the past two decades in support of Dr. Paul I. Terasaki's humoral theory of transplantation [1]. In kidney transplantation, the presence of donor-specific anti-human leukocyte antigen (HLA) antibodies has been associated with acute and chronic rejection, as well as impaired graft function and accelerated graft failure [2, 3]. Donor-specific HLA antibodies (DSA) cause vasculitis and rejection in cardiac allografts which contribute to graft dysfunction and poor clinical outcomes [4]. In lung transplantation, the emergence of de novo DSA has been linked with the bronchiolitis obliterans syndrome and inferior patient survival [5]. The presence of de novo DSA has also been identified as a strong independent predictor of allograft failure among pancreas transplant recipients [6].

The role of humoral alloreactivity in liver transplantation, on the other hand, remains unclear. Since the earliest days of experimental liver transplantation (LT), the liver has been recognized as an immunologically privileged organ with relative resistance to rejection [7]. The tolerogenic capacity of the liver graft is not limited to cell-mediated alloimmunity but also appears to extend to antibody-mediated inflammation as well. Several mechanisms have been proposed for the tolerogenic properties of the liver, and it is likely that multiple pathways act in concert to circumvent immunologic rejection [8]. One such theory revolves around the liver allograft's ability to secrete soluble HLA class I antigens [9]. Together with the plethora of cell-bound HLA class I antigens expressed within the liver, the organ has a tremendous ability to absorb or neutralize alloantibodies directed against HLA antigens [10]. Indeed, an estimated 85% of LT recipients with preformed alloantibodies will eliminate circulating DSA within the first few months after transplantation [11]. These mechanisms, however, do not confer complete protection against allospecific HLA antibodies; LT recipients who develop de novo DSA demonstrate inferior survival, particularly when DSA against HLA class II antigens [12–14] and IgG3 subclass DSA [15] are present at high titers. Other reports have associated DSA with late acute rejection [16] and chronic ductopenic rejection [17]. A summary of the recent studies investigating the effects of de novo DSA on LT outcomes is presented in Table 1.

Many questions remain with regard to the effects of alloantibodies on liver allografts. Are HLA antibodies a cause or consequence of liver injury? What are the histopathologic characteristics of antibody-mediated rejection in the liver graft? Why does the liver appear to resistant to antibody-mediated injury? Are all HLA antibodies pathogenic, and how do we predict which recipients with alloantibodies will progress to graft failure? In this article we will examine the available data pertaining to DSA in LT, and draw parallels to lessons learned from renal transplantation. We will also introduce novel perspectives and potential explanations for which the liver is less susceptible to injury mediated by HLA antibodies.

## 2. Known Effects of Alloantibody on the Liver Allograft

### 2.1. Acute Antibody-Mediated Injury

Demetris et al. have described two distinct histopathologic phenotypes associated with antibody-mediated rejection (AMR) in the liver graft, acute and chronic AMR [20]. Acute AMR is extraordinarily rare, occurring in less than 1% of all LT cases, and is almost exclusively limited to the first few weeks after transplantation in highly sensitized recipients [9, 21]. The few cases of acute AMR [22–29] reported among recipients of ABO-compatible LT in the era of solid-phase antibody testing are summarized in Table 2. Ischemia-reperfusion (IR) injury in the immediate posttransplant period activates the innate immune system and other nonimmune mechanisms, generating an inflammatory milieu that predisposes to allograft rejection. The clinical features of acute AMR resemble those seen with ABO-incompatible transplants and include allograft dysfunction, DSA persistence, refractory thrombocytopenia, and hypocomplementemia. Histopathologically, acute AMR is characterized by portal edema, endothelial cell hypertrophy, and eosinophilia within the portal microvasculature, hepatocyte swelling, ductular reaction, and cholestasis [20, 30]. These patterns of injury are analogous to findings indicative of capillaritis as seen with AMR of other solid organ allografts.

### 2.2. Mixed Cell-Mediated and Antibody-Mediated Rejection

In contrast to acute AMR, chronic AMR often encompasses features of both cellular and humoral immune reactivity. It is conceivable that, in moderate to severe cases of T-cell-mediated rejection (TCMR), helper T cells can activate humoral immunity and stimulate the production of alloreactive antibodies. In turn, donor-specific HLA antibodies can further potentiate the cellular alloimmune response, thereby increasing the severity of rejection. Compared with acute AMR which occurs in a small minority of sensitized recipients, this clinical picture of mixed TCMR/AMR is much more commonly encountered in actual practice.

In a study of 65 LT recipients presenting with acute allograft dysfunction, paired serum and tissue samples were obtained at the time of hospital admission. Of the 48 recipients with biopsy-proven rejection, donor-specific HLA class I and/or class II antibodies were identified by Luminex-based single antigen bead testing in 25 (52%) of recipients. The presence of strong class II DSA (mean fluorescence intensity ≥ 10,000) was associated with steroid-resistant rejection, as well as increased rejection severity. Furthermore, in the majority of cases DSA quickly diminished following resolution of the acute rejection episode, while the few remaining patients with persistent DSA were likely to progress to chronic rejection (unpublished data).

Similar findings were reported by Musat et al. [31], who retrospectively reviewed tissue and serologic data on 43 LT recipients presenting with graft rejection. The authors reported that diffuse portal C4d deposition, together with DSA positivity in the serum, predicted the frequency of acute rejection episodes, as well as the likelihood of steroid-resistant rejection and ductopenia. Taken together, these observations suggest that humoral alloreactivity is closely intertwined with cellular mechanisms during acute rejection; alloantibodies may be a direct consequence of cell-mediated immunity, but antibodies may in turn intensify the degree of tissue injury.

## 3. Humoral Alloimmunity and Chronic Liver Graft Rejection

A more interesting question pertains to whether HLA antibodies elicit insidious inflammation, fibrosis, and chronic rejection of the liver allograft. Addressing this question requires the prospective collection of serial biopsies and serum samples to document the association between circulating DSA and the histopathologic progression from inflammation to fibrosis. Until recently, this data has not been available as protocol biopsies and alloantibody testing are not routinely performed for LT recipients, and most studies in this subject area have been retrospective in nature. In a prospective study of 89 stable pediatric LT recipients, Varma et al. [32] obtained serial protocol biopsies and analyzed the tissue for evolution of inflammation and fibrosis. The authors found an association between class II DSA and portal inflammation, which over time predisposes to fibrosis progression in the portal areas.

The story with alloantibodies in LT is much more complicated, however, as not all recipients with donor-specific HLA antibodies will develop clinicopathologic evidence of graft injury. Preformed DSA is present in an estimated 13–17% of LT recipients [19], and an additional 8% will develop de novo DSA within the first year after transplant [13, 15]. Yet chronic rejection is an infrequent reason for allograft loss following LT, affecting only 3-4% of liver allografts among adult recipients maintained on tacrolimus-based immunotherapy [33]. Furthermore, results from immunosuppression withdrawal trials in pediatric LT recipients indicate that even operationally tolerant patients may harbor DSA, and the mere presence of DSA does not necessarily correlate with progressive increase in histologic inflammation or fibrosis [34].

## 4. Perspectives on the Liver Allograft's Resistance to Antibody-Mediated Injury

### 4.1. Incidence of Donor-Specific HLA Antibodies after Liver Transplantation

One of the potential explanations for the relative infrequency of AMR in LT is the lower incidence of DSA observed among LT recipients. Although up to 25% of LT candidates may be sensitized heading into transplantation, the vast majority of LT recipients clear all preformed DSA by four months after transplant [11]. The incidence of de novo DSA is approximately 5–14% after LT [13, 15, 18, 19], whereas de novo DSA has been reported in up to 28% of recipients following kidney transplantation [35]. The reasons for the apparently lower incidence of de novo DSA in LT have yet to be completely elucidated but may be attributable to the same mechanisms which confer tolerogenic properties to the liver, including the ability to absorb or neutralize alloantibodies [10, 36], as well as the immunomodulatory milieu imparted by nonparenchymal liver cells, including sinusoidal endothelial cells, Kupffer cells, resident dendritic cells, and hepatic stellate cells [8]. The unique structural and functional features which contribute to tolerance in the liver graft will be further explored below.

### 4.2. Unique Cellular Architecture of the Liver

The liver has several unique anatomic and immunologic features which renders its ability to avert antibody-mediated injury. First, the liver receives a dual blood supply from two distinct circulatory routes, from the systemic circulation via the hepatic artery and from the mesenteric system via the portal vein. On a microscopic level, hepatocytes are arranged in sheets surrounded by vascular sinusoids, which are enlarged capillaries lined by a fenestrated endothelium without an underlying basement membrane. Blood collected in these sinusoids drain into the central venule which then returns the blood to the systemic circulation.

The pathologic lesions indicative of AMR have been clearly defined in renal transplantation and are characterized by microvascular endothelial cell injury, as manifested by peritubular capillaritis, glomerulitis, and basement membrane duplication [3, 37, 38]. The distinct morphology of the liver sinusoidal endothelium, with a larger luminal diameter, fenestrated endothelium, and lack of a basement membrane may confer its resistance to microvascular damage. Accordingly, most of the histopathologic evidence of AMR in the liver has been observed in the small vessels with continuous endothelia such as the portal microvasculature, hepatic arterial capillaries, and the peribiliary plexus [39, 40], not within the hepatic sinusoids.

The peribiliary plexus is thought to be derived from hepatic arterial branches and appears exceptionally susceptible to ischemia and immunologic insults [41, 42]. Disruption of the peribiliary plexus by DSA causes arterial insufficiency which, in turn, incurs damage to the biliary epithelium and predisposes to the formation of bile duct strictures [43]. These mechanisms likely account for bile duct atrophy and ductopenia which are typically seen in late acute or chronic rejection [31, 44]. The temporal relationships between rejection, endothelial cell injury, and bile duct loss have been described by Matsumoto et al. [45] in comparing biopsies from rejecting and normal allografts. Both acute and chronic rejection are associated with a reduction in the number of portal microvascular structures, a finding most pronounced in severe cases of rejection. They further demonstrated that components of the microvasculature were destroyed prior to disappearance of the bile ducts. Taken together, the unique cellular architecture of the liver likely plays an important role in its resistance to antibody-mediated inflammation and fibrosis.

### 4.3. The Immunomodulatory Liver Parenchyma

The hepatic portal circulation is constantly exposed to microbial products and foreign antigens from the gut, and the liver has acquired a number of molecular adaptations to avoid unnecessary immune responses to innocuous antigens. Liver sinusoidal endothelial cells (LSECs), together with Kupffer cells and resident dendritic cells found within the hepatic sinusoids, play essential roles in the maintenance of immunologic tolerance. At steady state, the liver demonstrates strong, diffuse expression of class I major histocompatibility complex (MHC) antigens, but weak class II MHC expression. The normal liver also secretes soluble class I HLA molecules, which can complex with alloantibody and then become vigorously cleared by Kupffer cells in the hepatic sinusoids [46]. Additionally, antigen presentation by LSECs often leads to the release of anti-inflammatory cytokines and the preferential expansion of tolerogenic T-cell subsets, creating an immunomodulatory microenvironment within the liver.

This pattern of tolerance can be broken by pathogenic stimuli such as infectious microorganisms and endotoxin, as well as by endogenous damage-associated molecular patterns generated during preservation injury. Following inflammatory insults, class II MHC expression is upregulated on biliary epithelial cells, portal and hepatic artery endothelia, resulting in increased DSA targeting and further immune stimulation. An exhaustive review of the tolerogenic mechanisms and immune reactions within the liver is beyond the scope of this article, and the interested reader is encouraged to refer to an excellent review article written by Demetris et al. [41].

Because of the high tolerogenic threshold within the liver, humoral alloimmunity does not typically elicit tissue injury without coexisting insults. This is best illustrated by normal biopsies without histologic signs of tissue injury which can be obtained from clinically stable LT recipients with circulating DSA, even among patients who have undergone complete withdrawal of immunosuppression therapy [11, 47]. Based on this evidence, Kim et al. have proposed the “two-hit hypothesis,” in which a concurrent insult causing allograft inflammation is needed for alloantibodies to incur observable dysfunction [48].

Further support for the “two-hit hypothesis” originates from epidemiologic studies of chronic rejection among primary LT recipients [49]. Among patients receiving tacrolimus-based immunotherapy, the risk of chronic rejection is increased by the occurrence of acute cellular rejection, advanced donor age, hepatitis B or hepatitis C viral infections, and diagnoses of primary biliary cirrhosis, primary sclerosing cholangitis, or autoimmune hepatitis. Each of these etiologic factors introduces an alternate source of allograft injury, such as T-cell-mediated alloimmunity, augmented IR injury in a suboptimal donor organ, chronic active viral hepatitis, or autoimmunity. These insults tip the delicate immune balance within the liver towards a proinflammatory phenotype and may act in concert with alloantibodies to promote fibrosis and graft loss.

## 5. Implications and Conclusions

In comparison to other solid organ transplants, the liver allograft appears exceptionally tolerant to cellular and humoral alloimmune activity. While the unique anatomic and functional properties of the liver contribute at least in part to its tolerogenic capabilities, the protection conferred by these attributes is not complete. Alloantibody-mediated inflammation in the liver graft can occur, particularly in the presence of class II DSA and in the face of coexistent insults, which if left untreated will likely culminate in tissue injury and irreversible graft damage.

Because humoral alloimmunity is closely intertwined with other mechanisms of inflammation (such as cell-mediated alloimmunity and IR injury), the identification of unique histopathologic features indicative of antibody-mediated injury has been a particularly challenging task. C4d deposition, which is a specific marker for humoral activity in kidney transplantation, lacks the same degree of accuracy in liver allografts and can often be detected among recipients with nonrejection causes of dysfunction. In the 2016 update of the Banff Working Group on Liver Allograft Pathology, histopathologic criteria for the diagnosis of AMR was introduced for the first time [40]. Clearly more research efforts are needed to establish the link between humoral immune pathways and histopathologic features of antibody-mediated injury in liver grafts.

**Figure 1:**
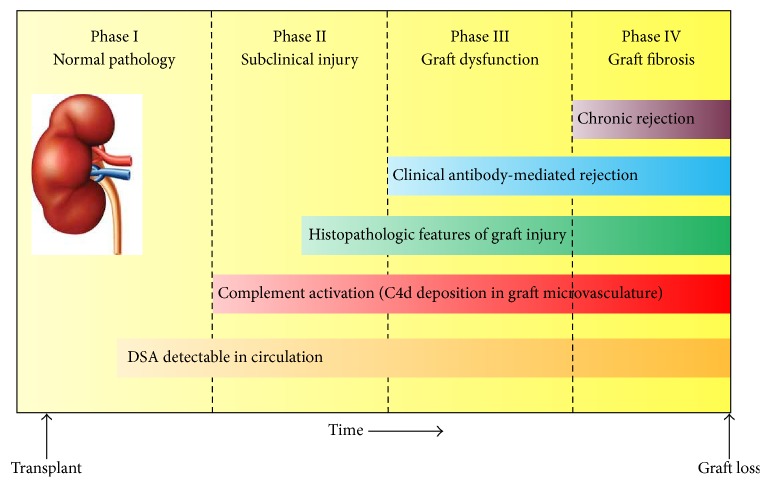
Natural progression of antibody-mediated rejection in renal transplantation. DSA, donor-specific antibody.

Kidney transplant experts have proposed a hypothetical chain of events (Figure 1) which describe the temporal progression of AMR, starting from the serologic presence of HLA alloantibodies (Phase I), to histopathologic evidence of tissue injury via C4d deposition (Phase II), to clinically evident graft dysfunction (Phase III) and irreversible graft fibrosis (Phase IV). Great efforts have been made to identify patients in the earlier phases of this sequence by routine serologic testing and protocol biopsies, in an attempt to hinder the humoral immune response before irreversible damage to the graft is incurred. Despite advances in DSA testing with the sensitive and specific Luminex-based single antigen testing, currently available antibody-reduction protocols have failed to demonstrate reliable and sustained eradication of DSA after transplant. Consequently, AMR and chronic rejection still remain as frequent causes for renal allograft loss.

**Figure 2:**
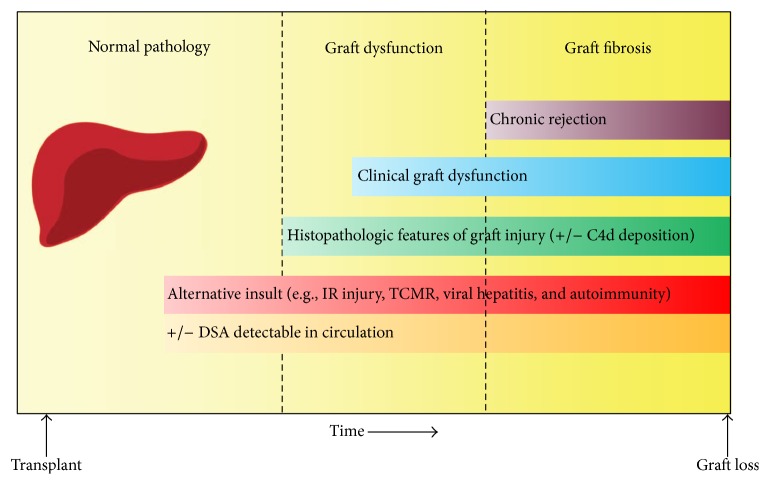
Proposed sequence of events leading to the development of chronic rejection in liver transplantation. DSA, donor-specific antibody; IR, ischemia-reperfusion; TCMR, T-cell-mediated rejection.

I herein propose a modified sequence of events for liver transplant recipients who harbor persistent DSA based on the evidence presented in this review (Figure 2). In many LT recipients, humoral alloimmunity requires a concomitant insult to overcome the immunoregulatory tendency of the liver allograft to induce tissue injury. Occasionally, the emergence of DSA may even be a direct result of these alternative sources of inflammation, such as stimulation from innate immune pathways and from T helper cells. Acting together, the coexisting insult and humoral immunity will quickly initiate allograft inflammation and clinically evident dysfunction. This model introduces significant implications for the monitoring and management of LT recipients.

1. Antibody-mediated injury can be ameliorated or prevented altogether by addressing the coexistent causes of allograft injury. Our treatment strategies for these alternative insults to the liver allograft have generally been much more effective than the protocols designed for antibody reduction. For instance, the activation of cell-mediated immunity can be avoided by adequate immunosuppression and by promoting medication adherence. IR injury can be minimized by appropriate donor selection and minimization of ischemia times, and a great deal of research efforts have been dedicated to identifying strategies that attenuate IR injury following transplantation. Impressive advances have been made in the treatment of hepatitis B and hepatitis C virus infections over the past few decades, and sustained viral responses can be consistently achieved to prevent viral reinfection after LT [50].
2. Routine posttransplant monitoring for HLA antibodies is not necessary for all LT recipients but may be beneficial for selected patients with coexisting reasons for graft inflammation, such as those with diagnoses of autoimmune liver diseases or acute TCMR. This recommendation is based on the premise that alloantibodies require an alternative insult or a “second hit” to incur tissue injury. Testing for DSA in these subgroups of patients may help identify those at highest risk for severe graft damage and accelerated graft loss.

Knowledge gaps remain pertaining to the immunologic mechanisms leading to liver allograft failure. Given the relative immunologic ease of managing LT recipients and the small percentage of grafts lost to chronic rejection, transplant professionals have historically focused on more urgent issues such as the critical donor organ shortage, recurrent diseases after transplantation, and complications from the long-term use of immunosuppressive drugs. However, given recent trends in LT, including the rising use of marginal donor organs, advances in the treatment of recurrent diseases, and attempts to withdraw or minimize immunosuppression, we are likely to witness an increase in the immunologic consequences of humoral reactivity. More work is needed to decipher the complex interactions between the humoral immune system and the liver allograft and to identify the contexts in which alloantibodies incur tissue injury following transplantation.

## Figures and Tables

**Table 1:** Summary of recent studies of de novo DSA on clinical outcomes ABO-compatible liver transplantation.

Reference	Study design	Sample size	Prevalence of de novo DSA	Study findings
Kaneku et al. (2013) [13]	Retrospective	749 adult	8.1% at 1 year	(i) Presence of DSA associated with inferior patient and graft survival (ii) Almost all de novo DSA were against HLA class II antigens (majority DQ) (iii) Risk of de novo DSA formation increased by low calcineurin inhibitor levels and the use of cyclosporine (versus tacrolimus)

Grabhorn et al. (2015) [17]	Retrospective	43 pediatric	33% in stable recipients; 68% in chronic rejectors	(i) Higher rate of de novo DSA among pediatric LT recipients with chronic rejection (ii) Antibodies predominantly against HLA class II antigens

O'Leary et al. (2015) [15]	Retrospective	749 adult	8% at 1 year	(i) IgG3 subclass DSA-positive patients at highest risk for death (ii) IgG3-negative, DSA-positive patients still had inferior outcomes compared to DSA-negative patients

Wozniak et al. (2015) [16]	Cross-sectional	50 pediatric	54%	(i) Younger age associated with presence of DSA (ii) Nontolerant patients more likely to have DQ DSA (61%) compared with stable (20%) and tolerant (29%) patients (iii) DQ DSA associated with de novo autoimmune hepatitis and late acute rejection

Del Bello et al. (2015) [18]	Prospective	152 adult	14%	(i) Younger age, low exposure to calcineurin inhibitors, and noncompliance were risk factors for de novo DSA emergence (ii) Nine of 21 (43%) DSA-positive recipients developed acute rejection (iii) No differences in patient or graft survival with DSA presence

Levitsky et al. (2016) [19]	Retrospective analysis of an observational cohort study	195 adult (129 LDLT, 66 DDLT)	5.4% in LDLT; 6.1% in DDLT	(i) No differences in the prevalence of de novo DSA between LDLT and DDLT recipients (ii) Presence of DSA was an independent risk factor for graft failure in LDLT and DDLT

LDLT, living donor liver transplantation; DDLT, deceased donor liver transplantation.

**Table 2:** Summary of reported cases of acute antibody-mediated rejection following ABO-compatible liver transplantation.

Reference	Age/gender	Onset of graft dysfunction	DSA detection method	Type of DSA	DSA specificity	AMR treatment	Clinical outcome
Rostron et al. (2005) [22]	23/F	POD 6	Luminex SAB	Preformed	Bw6	Steroids, MMF, PP, IVIG	Alive with functioning graft

Wilson et al. (2006) [23]	36/F	4 years	Luminex SAB	De novo	DR52	Steroids, MMF, PP, IVIG, Rituximab, ATG	Alive with functioning graft

Watson et al. (2006) [24]	50/F	POD 5	Flow cytometry SAB	Preformed	B7	Steroids, MMF, PP, IVIG, Rituximab	Death

Kamar et al. (2009) [25]	49/F	POD 10	Luminex SAB	Preformed	A2, DR7	Steroids, MMF, PP, Rituximab, OKT3	Death
	39/F	POD 6	Luminex SAB	Preformed	A2, A24, B27, DR4	Steroids, PP, Rituximab	Alive with functioning graft

Kozlowski et al. (2011) [26]	N/A	POD 5	Flow cytometry or Luminex SAB	Preformed	3 DSA (specificity not provided)	Steroids, PP, IVIG, Rituximab	Death
	N/A	POD 7		Preformed	A30, A74, B7, B45, DR15, DR51, DQ7	Steroids, PP, IVIG, Rituximab, ATG	Death
	N/A	POD 7		Preformed	4 DSA (specificity not provided)	Steroids, PP, IVIG, Rituximab, OKT3	Retransplant, alive

Paterno et al. (2012) [27]	62/F	POD 8	Luminex SAB	De novo	DR13, DR15, DR51, DR52	Steroids, OKT3, ATG, Bortezomib	Alive with functioning graft
	28/F	POD 452	Luminex SAB	De novo	DQ2, DQ6	Steroids, PP, Rituximab, ATG, Bortezomib	Alive with functioning graft
	53/F	POD 6	Luminex SAB	Preformed	B51, Cw2, DQ7	Steroids, ATG, Bortezomib	Alive with functioning graft

Kheradmand et al. (2014) [28]	43/F	POD 1	Luminex SAB	Preformed	B35, B51, DR4, DR53, DQ8	Steroids, PP, IVIG, Rituximab, ATG	Alive with functioning graft

Wozniak et al. (2016) [29]	22 mo/M	POD 45	Luminex SAB	De novo	B44, DQ2	Steroids, MMF, IVIG, Rituximab	Alive with functioning graft
	3/F	POD 13	Luminex SAB	N/A	A1, DQ5	Steroids, MMF, IVIG	Alive with functioning graft
	19 mo/F	POD 8	Luminex SAB	Preformed & de novo	Cw7, Cw17, DR4, DR53, DQ8	Steroids, MMF, IVIG, Rituximab	Alive with functioning graft
	11/M	POD 7	Luminex SAB	De novo	DR53, DQ8	Steroids, MMF, PP, IVIG, ATG, Bortezomib	Alive with functioning graft
	6 mo/M	POD 38	Luminex SAB	De novo	DQ7, DQ9	Steroids, MMF, PP, IVIG, Rituximab	Retransplant, death
	3/M	POD 7	Luminex SAB	Preformed	A1, B8, Cw7, DR17, DQ2, DP1	Steroids, MMF, PP, IVIG, Rituximab, Bortezomib, Eculizumab	Alive with functioning graft

N/A, not available/information not provided; SAB, single antigen bead-based testing; PP, plasmapheresis; IVIG, intravenous immunoglobulin; ATG, antithymocyte globulin.

## Abbreviations

AMR: Antibody-mediated rejection
DSA: Donor-specific antibody
HLA: Human leukocyte antigen
IR: Ischemia-reperfusion
LSEC: Liver sinusoidal endothelial cell
LT: Liver transplantation
MHC: Major histocompatibility complex
TCMR: T-cell-mediated rejection.
